# A comprehensive review on clinical and mechanistic pathophysiological aspects of COVID-19 Malady: How far have we come?

**DOI:** 10.1186/s12985-021-01578-0

**Published:** 2021-06-07

**Authors:** Baila Shakaib, Tanzeel Zohra, Aamer Ikram, Muhammad Bin Shakaib, Amna Ali, Adnan Bashir, Muhammad Salman, Mumtaz Ali khan, Jamil Ansari

**Affiliations:** 1 Rawal Institute of Health Sciences, Islamabad, Pakistan; 2 National Institute of Health, Islamabad, Pakistan; 3 CMH Medical College, Lahore, Pakistan

**Keywords:** Pathology, COVID-19, Manifestations, Vaccine, Entry mechanism

## Abstract

Since its outbreak in 2019, the coronavirus disease (COVID-19) has become a pandemic, affecting more than 52 million people and causing more than 1 million mortalities globally till date. Current research reveals a wide array of disease manifestations and behaviors encompassing multiple organ systems in body and immense systemic inflammation, which have been summarized in this review. Data from a number of scientific reviews, research articles, case series, observational studies, and case reports were retrieved by utilizing online search engines such as Cochrane, PubMed, and Scopus from December 2019 to November 2020. The data for prevalence of signs and symptoms, underlying disease mechanisms and comorbidities were analyzed using SPSS version 25. This review will discuss a wide range of COVID-19 clinical presentations recorded till date, and the current understanding of both the underlying general as well as system specific pathophysiologic, and pathogenetic pathways. These include direct viral penetration into host cells through ACE2 receptors, induction of inflammosomes and immune response through viral proteins, and the initiation of system-wide inflammation and cytokine production. Moreover, peripheral organ damage and underlying comorbid diseases which can lead to short term and long term, reversible and irreversible damage to the body have also been studied. We concluded that underlying comorbidities and their pathological effects on the body contributed immensely and determine the resultant disease severity and mortality of the patients. Presently there is no drug approved for treatment of COVID-19, however multiple vaccines are now in use and research for more is underway.

## Background

In late December 2019, a type of pneumonia with influenza-like symptoms (fever, dry cough, and fatigue) was reported in Wuhan, China [1]. The disease, caused by the betacoronavirus SARS-CoV-2, was later termed as the Coronavirus disease 2019 COVID-19. Declared a pandemic in March 2020 by the World Health Organization (WHO) [2], the disease has affected 52 million individuals worldwide with more than 1 million mortalities at the time of this review. Presently, there is no definitive drug regimen to treat SARS-CoV-2 infection and vaccines are either in trial phases or approved for limited use only [3, 4]. Thus, infection prevention and control methods such as avoiding mass gatherings, obeying social distancing protocols and observing strict personal hygiene regimens such as wearing facemasks, washing of hands and using hand sanitizers remain important components of disease prevention and management [5].

Another major component of disease management is timely detection [6] and resultant supportive care. Unfortunately, a large percentage of COVID-19 patients get themselves days after the onset of symptoms while many never do due to a number of reasons; either because they are asymptomatic or due to inaccessibility and low awareness regarding the disease [7]. This therefore, calls for a deeper understanding and constant revision of knowledge of the disease presentation and course through continued scientific research.

Initially, the disease was believed to behave similarly to acute respiratory distress syndrome (ARDS) or other viral illnesses with symptoms such as cough, shortness of breath, sore throat, rhinorrhea, hemoptysis, chest pain, diarrhea, nausea and vomiting, myalgia and fatigue, headache and confusion. However, the immense research carried out by the scientific community across the globe shows that the disease is reported to have a much wider range of clinical manifestation; starting from complete absence of symptoms to mild ones mimicking typical flu-like symptoms to the more aggressive symptoms such as multiple organ involvement. The latter includes but not limited to the cardiovascular, renal, cerebrovascular, gastrointestinal, dermatological and ocular systems. The pandemic continues to affect millions globally since its first outbreak approximately a year ago and multiple studies regarding different aspects of the disease have been researched upon during the entire course of the on-going global outbreak. We present a comprehensive review of studies regarding clinical presentation of the disease described in the literature till writing.

## Methodology

### Search strategy and data selection

A comprehensive literature search and analyses were conducted to include data published from December 2019–November 2020 utilizing online search engines Cochrane, PubMed, Scopus and Google scholar. Thirteen original researches were included for pooled analyses of disease characteristics by considering sample size, impact factor and disease variables. Studies covering a wide range of age groups (18–90 years of age) were included. Data from Studies focused on a particular age group were not included for the pooled analyses, but mentioned separately in addition to the pooled analyses. Studies published in English or having a translated version in English were included. Studies published in Chinese or any other language and not having a translated version were excluded. Studies focused and presenting symptoms from only one organ system were excluded from the pooled analyses, but mentioned otherwise in the article (see “ocular manifestations” section below). Systematic reviews, cohort studies, cross-sectional studies, case series and case- reports were also included. Studies from different parts of the world—China, USA, Europe and Southern America—were included. Studies for both adults and children were included and discussed under in separate sections of the paper. A string for the literature search has been provided as a supplementary material.

### Data analysis

EndNote X8.0 software was used to manage, record and omit duplicates. Raw data from thirteen original studies which reported signs and symptoms were compiled and analyzed using SPSS software version 25. Data were analyzed and displayed using median % (interquartile range) as a measure of central tendency. For a particular sign, if the study had not recorded it, the total count of the study was deducted from the original pool for that particular variable. Signs and symptoms for all organ systems were recorded separately. Variables from case reports were discussed separately.

The underlying pathophysiological mechanisms for certain disease symptoms were illustrated using an online software BioRender.

#### Disease signs, symptoms and organ involvement

The disease is generally classified as asymptomatic, mild, moderate, severe, and critical which includes both involve intensive care unit (ICU) and mortality (Table 1). Presence of underlying comorbidities, such as diabetes, cardiovascular, and cerebrovascular diseases can worsen disease prognosis [8, 9].

**Table 1:** Summarizes frequency of signs and symptoms by means of median and interquartile range (IQR) recorded for COVID-19 patients for studies included in the review

Organ system	Symptom	Frequency (%)		Studies reported
		Median	IQR	
Cerebrovascular	Headache	8.2%	7.1–16.0	7
	Confusion	9.1%	–	1
	Dizziness	9.4%	–	1
Eye*	–	–	–	0
Ear, Nose, Throat	Nasal Congestion/ Rhinorhea	16.7%	6.9–61.5	4
	Sore throat	11.1%	5.9–16.5	4
	Sputum Production	30.2%	10.0–39.7	4
	Tonsil swelling	2.0%		
Chest/Pulmonary	Cough	71.4%	46.7–73.1	12
	Shortness of breath	36.7%	19.0–56.5	11
	Hemoptysis	4.9%	1.0–5.1	4
	Chest pain	2.0%	–	1
	Chest distress	23.8%	–	1
Gastrointestinal	Loss of appetite	39.9%	12.2–49.5	3
	Nausea/ Vomiting	12.7%	4.0–19.5	6
	Diarrhea	8.0%	3.2–11.8	10
	Abdominal pain	4.4%	4.3–5.8	3
Musculoskeletal	Fatigue	66.9%	49.9–73.6	4
	Myalgia/Arthralgia	23.1%	14.6–33.5	9
Dermatological	Rash	0.2%		1
*Eye	None of the original searches contained data on ocular signs and symptoms. A cohort [57], however, focused on ocular signs and symptoms revealed: eye pain (19.4%), photophobia (13.9%), flashes or floaters (11.8%), blurring of vision (11.1%), and eye redness (10.4%)			

Disease severity classification	Features (as per CDC guidelines)			
Asymptomatic/presymptomatic	Patients who test positive for SARS-CoV-2 but have no symptoms suggestive for SARS-cov-2 infection			
Mild Illness	Patients having any of the various signs or symptoms of SARS-CoV-2 infection but do not have shortness of breath, dyspnea or abnormal chest imaging			
Moderate Illness	Individuals who show signs of lower respiratory disease on clinical assessment or imaging and have an oxygen saturation (SpO_2_) of ≥ 94% on room air at sea level			
Severe Illness	Individuals with SpO2 < 94% on room air at sea level, a ratio of arterial partial pressure of oxygen to fraction of inspired oxygen (PaO2/FiO2) < 300 mm Hg, respiratory frequency > 30 breaths/min, or lung infiltrates > 50%			
Critical Illness	Individuals having respiratory failure, septic shock, and/or multiple organ dysfunction			

The most common manifestation was cough, followed by fatigue. Shortness of breath, chest distress and chest pain were most prevalent among cases exhibiting severe symptoms. There were no ocular symptoms recorded for any of the studies included in this review

Patients develop symptoms of COVID-19 infection following a mean incubation period of 5.2 days (95% CI, range: 4.1–7.0) [10]. The time period between development of COVID-19 symptoms and death varies from 6 to 41 days (median:14 days) [11], depending on the patient’s age and immune status. It is shorter for patients older than 70 years of age than for patients under 70 years of age [11] as the disease affects the elderly more severely [12]. COVID-19 most frequently presents with fever, cough, and fatigue. Other symptoms include headache, sputum production, hemoptysis, dyspnea, diarrhea, and lymphopenia [13–15]. An estimated 67% (40.1–91.7) of patients in the review had fever (rest of the symptoms will be discussed later in association with relevant organ systems). Research shows that males, especially older, are more affected than females [16]. In the present analyses, mean percentage of male patients was 59.0% (SD 10.0). A recent study, focusing on patients suffering from mild to moderate symptoms, who had not received immunomodulatory treatment, revealed that males had higher levels of innate immune cytokines and chemokines including IL-8, IL-18, and CCL5 in their plasma, along with stronger induction of non-classical monocytes. Conversely, females manifested significantly more robust activation of T-cells than males during SARS-CoV-2 infection, which was sustained in old age. It also showed that a poor T-cell response was negatively correlated with patient’s age and predisposed primarily males to worse disease outcomes. In contrast, higher innate immune cytokines in only females predicted worse disease progression[16].Disease severity and mortality are known to vary across different populations due to variations in a wide array of biological and environment factors as listed below such as:

i. Genetic variations in Human Leucocyte Antigens (HLA) and the resultant resulting immune response[17, 18]
ii. ACE2 allelic variability[19]
iii. ABO blood groups, A has a higher susceptibility (no established correlation with disease severity) to COVID-19 ( OR, 1.45; 95% CI, 1.20—1.75; *p* = 1.48 × 10^−4^) while O has a protective effect (OR, 0.65; 95% CI, 0.53—0.79; *p* = 1.06 × 10^−5^))[20]
iv. Genetic susceptibility (3p21.31 gene cluster in COVID-19 patients with respiratory failure) [21]
v. The protective effect of BCG vaccine and fewer COVID-19 cases in a country with universal policies of BCG vaccination [22]
vi. Ethnicity (black population has a higher predisposition to COVID-19) [7]
vii. Environmental factors such as temperature and humidity with the unit increase resulting in decreased COVID-19 mortality [23],
viii. Socioeconomic status[7].

Initially considered to present mainly with pulmonary symptoms, COVID-19 has shown to involve multiple organ systems along with nonconventional symptoms showing extra-pulmonary involvement. [24].

### Head and neck

#### Cerebrovascular disease and neurological symptoms

About 36% of COVID-19 cases manifest neurological symptoms during acute phase of illness. An estimated 25% of these cases have shown direct central nervous system (CNS) involvement. The most frequently reported neurological symptoms include dizziness, headache, impaired consciousness and seizures [25], with or without a history of pre-existing neurological disorders. At the time of hospital discharge, one-third of the patients have manifested motor defects and cognitive impairment [26]. Patients have shown confusion, agitation and corticospinal tract abnormalities (such as enhanced tendon reflexes and clonus) while being admitted in ICUs. In patients with mild to moderate disease, olfactory (85.6%) and gustatory (88.0%) dysfunctions have also been reported. Importantly, in about 11% of patients, anosmia was reported as the presenting symptom of the illness [27].

Table 1 summarizes the cerebrovascular symptoms recorded in this review. Headache was the most commonly reported cerebrovascular symptom and experienced by a median of 8.2% (7.1–16.0) of the patients.. Additionally, studies have reported sub-acute occurrence of the Miller-Fisher [28] and the Guillain-Barré syndromes [29] approximately 3 to 10 days after the onset of COVID-19 symptoms.

Cerebrovascular disease has been reported as an underlying condition in people with COVID-19 [30, 31]. Studies have reported large vessel stroke in young [32] and acute stroke in four cases of COVID-19 as presenting features of the disease [33]. A study suggests that T-cell hyper activation leads to paralysis which can result in pulmonary damage, cytokine release syndrome (CRS) and organ failure in individuals with severe COVID-19 disease [34]. Additionally, elevated D-dimer levels and markedly reduced platelets in critically ill patients can lead to acute cerebrovascular complications [35].

### The different mechanisms of cerebrovascular involvement

**Fig. 1:**
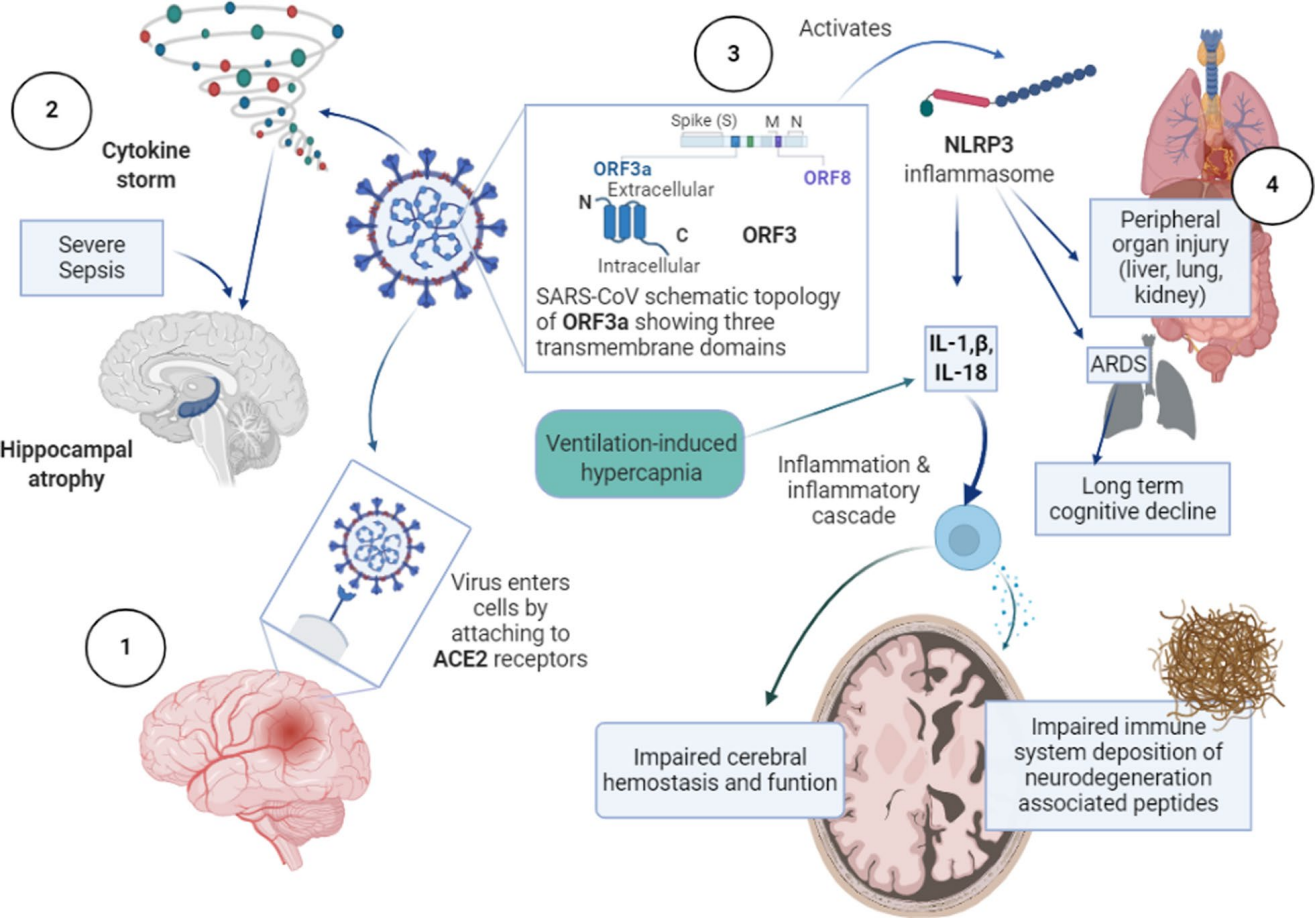
SARS-CoV-2 causes damage to the nervous system via 4 possible pathways: (1) direct viral encephalitis which caused by direct damage to brain tissue by SARs-CoV-2 as it enters brain cells through ACE2 receptors and causes inflammation and damage. The S (spike) proteins that cover the surface of the virus bind to ACE2 receptors and facilitate viral entry. Once the S protein binds to ACE2 receptor, TM protease serine 2 (TMPRSS2) located in the host cell membrane further facilitates virus entry by activating the S protein. After the virus has entered the host cell, viral RNA is released and protein cleavage and assembly of replicase-transcriptase lead to transcription and replication of viral genome [39]. (2) Systemic inflammation: SARS-CoV-2 infection elicits cytokine storm in the body which, along with severe sepsis can lead to Hippocampal atrophy. (3) cerebrovascular changes: the viral protein ORF3 and ventilation induced hypoxia activate inflammasome NLRP3 which can lead to increase in inflammatory cytokines especially IL-19 IL-1B which can trigger the inflammatory cascade causing impairment of immune system of the brain leading to deposition of pathological fibrillary tangles in brain tissue, and impatient of cerebral hemostasis and function[40]. (4) peripheral organ damage due to systemic inflammation and direct viral infection via ACE2 receptors and ARDS can lead to long term cognitive decline [36] (Image created with BioRender.com)

According to Heneka et al. [36], neurological signs and symptoms of COVID-19 may result from systemic inflammation, direct viral encephalitis, peripheral organ (liver, kidney, lung) dysfunction and cerebrovascular changes. Figure 1 describes these mechanisms in detail. This may be supported by the fact that firstly, SARS-CoV2 could induce cerebrovascular disorders including encephalitis, polyneuropathy and aortic ischemic stroke [37]; and secondly, the structure of SARS-CoV-2 is comparable to that of SARS-CoV (79.5%), and bat coronavirus (96%) [38]. Any one, or a combination of these pathological pathways, can predispose COVID-19 survivors to long-term neurological complications, either by exacerbating a preexisting neurological disorder or by triggering a new one [36].

Evidence suggests that activation of NLRP3 inflammasome contributes to lung injury, pathogenesis and adverse outcomes of ARDS [41]. In COVID-19 patients’ levels of interleukins, especially interleukin-1β and interleukin-18, are high which may be attributable to activation of NLRP3 inflammasome by coronavirus protein ORF3a [42], and ventilation-induced hypercapnia [43]. These processes and subsequent increased activation of pro-inflammatory pathways may disrupt cerebral homeostasis and function. Moreover, NLRP3 inflammasome-mediated inflammation has been linked to accumulation of neurodegeneration-associated peptides–such as fibrillar amyloid-β [44], which may aggravate neurodegeneration and progression of Alzheimer’s disease [40, 45, 46].

The systemic inflammation [47, 48], ARDS [49], and chronic ventilation [50, 51] in COVID-19 patients may cause short-term and long-term cognitive decline and neurodegenerative changes. Moreover, the presence of SARS-CoV-2 in the cerebrospinal fluid [26] has been confirmed and viral encephalitis and direct viral infiltration of the brain (retrograde neuronal dissemination or hematogenous spread) have been reported [52].

Furthermore, due to mass hysteria, social dysfunction resulting from self-isolation and quarantine, xenophobia, disease-associated stigma, economic effects and disrupted work-related and academic activities, an increased sense of anxiety and restlessness is evident in large groups of people [53, 54]. Ahmed et al. [55] also noted that young people (aged 21–40 years) were more likely to succumb to mental health conditions and alcohol use. Provision of direct patient care, vicarious trauma, quarantine, or self-isolation during the COVID-19 pandemic may cause health care providers to experience psychological distress [56].

#### Ocular signs and symptoms

None of the original researches included in this review had reported ocular symptoms. However, according to a recent cohort, eye pain (19.4%), photophobia (13.9%), flashes or floaters (11.8%), blurring of vision (11.1%), and eye redness (10.4%) have been the most frequently experienced ocular symptoms. The study found that ocular symptoms preceded the onset of systemic symptoms in only 20.6% (14/68) of cases. Twenty-six and a half percent (18/68) of respondents experienced persistent ocular symptoms despite recovery from systemic illness. Fifty-four percent (164/306) of COVID-19-negative patients reported experiencing at least one ocular symptom, thereby establishing no significant association of the presence of ocular symptoms with COVID-19. Symptoms experienced were not significantly different for COVID-19 positive and negative patients. Amongst COVID-19-negative, survey respondents, red eye (21.9%) and excessive tearing (17.6%) were found at a significantly higher rate [57].

A study from China [58] revealed that patients with COVID-19 who tested positive through a nasopharyngeal swab experienced ocular symptoms indicative of conjunctivitis (including conjunctival hyperemia, chemosis and epiphora), and increased secretions. It was also established that patients with ocular symptoms were more likely to have higher leukocyte count and neutrophil counts, and higher levels of procalcitonin, C-reactive protein (CRP), and lactate dehydrogenase (LDH) than patients who did not report any ocular symptoms. Additionally, ocular symptoms were reportedly more common amongst patients with severe disease than those with mild or moderate disease. Two out of eleven, patients who tested positive with nasopharyngeal swab also had positive RT-PCR results with conjunctival swabs, thereby suggesting the eyes as a possible source of disease transmission. A case of acute kerato-conjunctivitis as the presenting feature of COVID-19 has also been reported [59]. Besides the aforementioned information,, a child was reported to present with conjunctivitis and eyelid dermatitis as the only presenting signs of COVID-19 [60]. Other reports have also noted conjunctivitis as the first [61, 62], or the sole presenting symptom of COVID-19 [63].

Since a lot of ophthalmic procedures require close contact and can cause aerosol transmission, the ophthalmic community has been posed with great dilemmas and challenges with regards to clinical and surgical practices [64].

#### Ear, nose, and throat

Amongst the earliest symptoms of COVID-19, pharyngodynia has been common (12.4%), nasal congestion not as frequent (3.7%), and rhinorrhea quite rare [65]. Cases with Tonsil swelling [30] and sputum production [13, 66] have also been presented. Table 1 summarizes the symptoms studied in this review. Sputum production was the most common symptom recorded affecting 30.2% (10.0–39) of the patients. Anosmia, also as sudden new-onset symptom [67, 68], and hyposmia have been reported in COVID-19 patients [69].

Nasal discharge from a patient with post-viral olfactory dysfunction has already shown presence of human coronavirus 229E [70]. Like SARS-CoV, SARS-CoV-2 enters through ACE2 receptor [71].

### Chest and pulmonary

Most common chest and pulmonary signs and symptoms associated with COVID-19 are cough [30, 72], chest pain [73], chest distress/tightness [74], shortness of breath [13, 30], expectoration [75] and hemoptysis[13, 30].

Chest and pulmonary signs and symptoms studied in this review are also highlighted in Table 1. The most frequently experienced pulmonary symptom was cough [71.4% (46.7–73.1)] followed by shortness of breath [36.7% (19.0–56.5]), which has concurrently also been associated with disease severity [73].

**Fig. 2:**
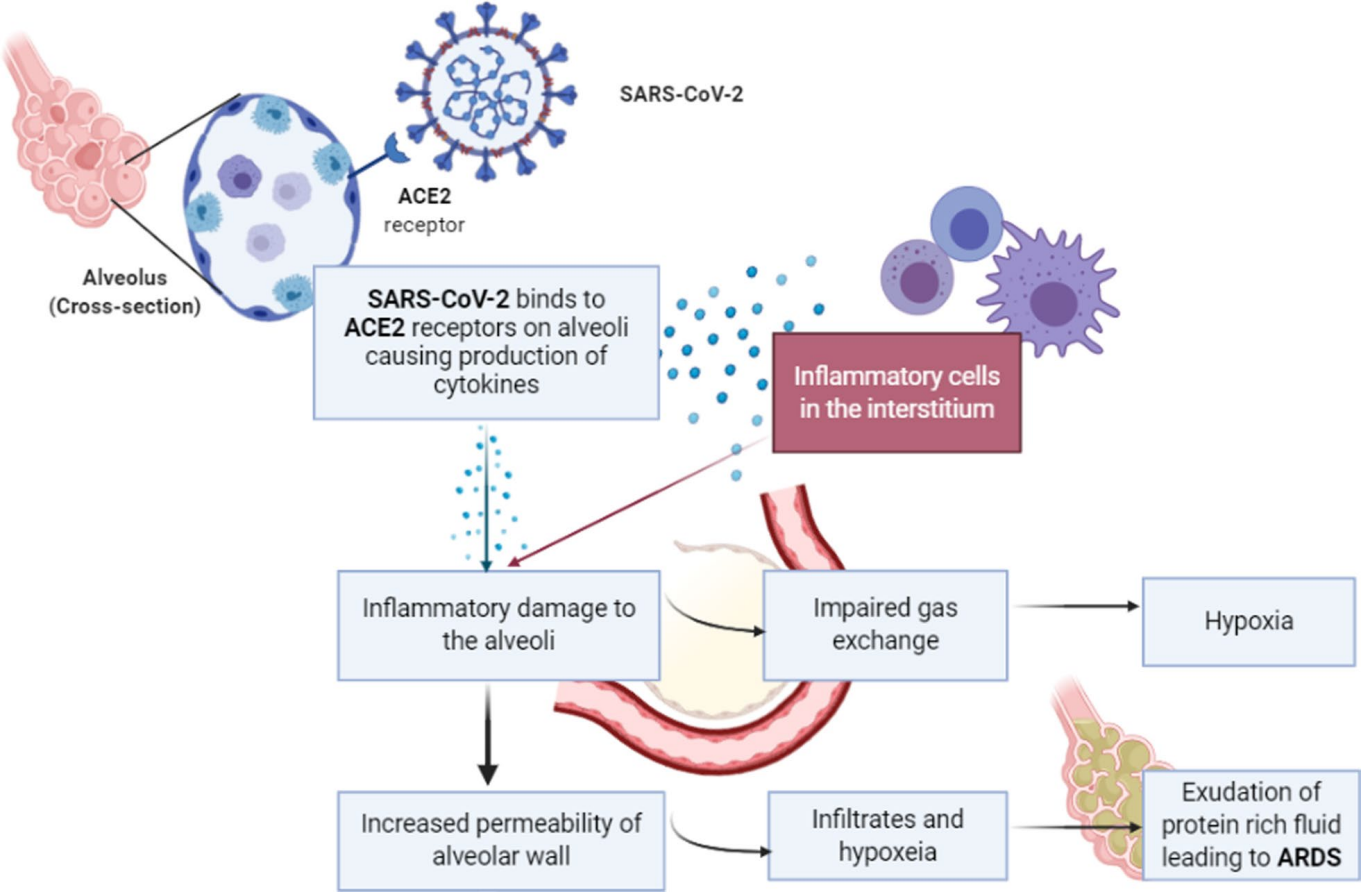
SARS-CoV-2 enters alveolar cells via ACE2 receptor. Initially, epithelial cells infected by SARS viruses act as a source of cytokines, releasing interleukin-1, interleukin-6, interleukin-8 and several other chemokines that can activate macrophages, attract neutrophils cause homing of monocytes and macrophages to sites of inflammation. After generating initial inflammatory response to alveolar damage, monocytes and macrophages are believed to contribute to production of cytokines. Thus, SARS-CoV triggers macrophages to increase production of certain chemokines (for example, MIP1α), and TNF and interleukin-6. This further activates neutrophils and macrophages which infiltrate the alveoli and cause tissue destruction [81]. (Image created with Biorender.com)

The pulmonary features of COVID-19 consist of diffuse alveolar damage and focal reactive hyperplasia of pneumocytes with patchy inflammatory cell infiltration (mostly monocytes and macrophages), vasculitis, hypercoagulability, and intravascular thrombosis [76, 77]. As a result, alveolar gas exchange is reportedly compromised (Fig. 2). The lungs of COVID-19 patients have high levels of inflammatory cytokines (IL-1, TNF) which are strong inducers of HA-synthase-2 (HAS2) in CD31^+^ endothelium, EpCAM^+^ lung alveolar epithelial cells, and fibroblasts [78]. Recent autopsies of COVID-19 patients have shown lungs filled with clear liquid jelly as can be seen in the case of wet drowning [79]. Although the composition of the jelly is yet to be determined, SARS infections have shown defective production of hyaluronan (HA), which is also associated with ARDS [80]. Patients with COVID-19 who require hospitalization and intensive care (ICU) have severe pneumonia with sub-acute hypoxic respiratory failure leading to ARDS, which can further precipitate shock– manifested as fever, lymphopenia, highly elevated proinflammatory cytokines, C-reactive protein (CRP), serum ferritin, and D-Dimers.

### Cardiovascular system

Cardiac injury is common among hospitalized COVID-19 patients [82, 83]. One-fifth of the hospitalized patients have been known to develop significant cardiovascular disease (manifested as troponin elevation, reduced ejection fraction, tachyarrhythmias and thromboembolic events), which is significantly linked with increased risk of mortality [84, 85]. In comparison to those without any apparent cardiac injury, patients with cardiac injury have been found to present with more severe acute illness, indicated by abnormal laboratory findings (such as higher levels of CRP, NT-proBNP, and creatinine levels) and radiographic findings (higher levels of multiple mottling and ground-glass opacities), and a greater need for either non-invasive or invasive ventilation.

COVID-19 patients have been reported to present with new-onset signs and symptoms of cardiac injury. A 64-year lady, with hypertension and dyslipidemia presented with chest pain and ST elevation on ECG and no fever or pulmonary symptoms [86]. Cases of COVID-19 patients presenting with cardiogenic shock and decompensated heart failure have also been reported [86]. A classic case of Kawasaki disease was reported in a 6-month-old child with COVID-19 infection [87].

Additionally, low platelet count has been shown to increase risk of severe disease and mortality in patients with COVID-19 and thus should indicate worsening illness during hospitalization [88]. One study also reported hypercoagulability along with clinical findings of pulmonary embolism and/or deep vein thromboses of the lower limbs in some COVID‐19 patients [89].

### Gastrointestinal tract manifestations

Since the outbreak of the disease, diarrhea has been considered an indicator of gastrointestinal (GI) tract involvement in COVID-19 patients (11.3%) [1]. The GI symptoms have been summed up in Table 1 [31, 74, 90]. Loss of appetite or decreased appetite, was the commonest symptom recorded for 39.9% (12.2–49.5) of the participants [74].

Han et al. found that COVID-19 patients experiencing digestive symptoms were likely to have a longer disease course between onset of symptoms and viral clearance than those who experienced only respiratory symptoms [73]. Additionally, patients with digestive symptoms took longer to seek medical care as noted by Pan et al. [91] which suggests delay in diagnosis resulting from delayed recognition of the disease.

Moreover, patients with digestive symptoms have been shown to have higher levels of liver enzymes, lower monocyte count, longer prothrombin time, longer time from onset of symptoms to admission, and receive more antimicrobial treatment than those without these symptoms [91].

**Fig. 3:**
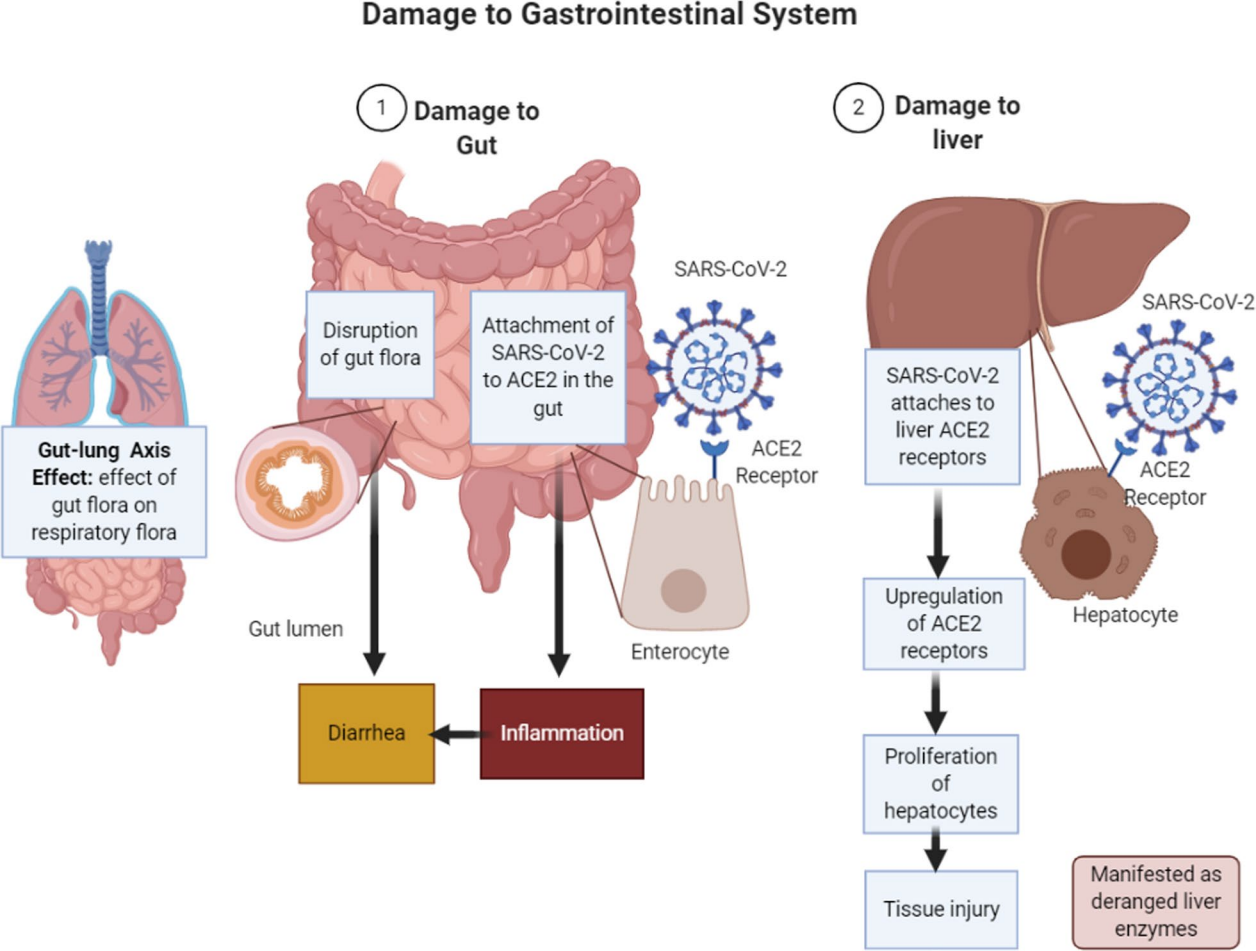
SARS-CoV causes damage to the gastrointestinal system through direct viremia (attachment to ACE2) receptors and inflammation. (1) In the gut, the virus binds to ACE2 receptors in the gut epithelium and enters the cells causing inflammation and damage to the epithelial cells. This can result in function of the epithelial cells and cause diarrhea [94]. Furthermore, it can disrupt the normal population of gut flora which has some important functions in the body including maintaining a balance between different organisms in the gut. SARS infection can disrupt this balance leading to diarrhea. Since the gut flora also has impact on respiratory flora and vice-versa, referred to as the gut lung axis, this also affects the flora of respiratory tract and hence lead to respiratory symptoms [95, 97]. (2) In the liver, viral attachment to ACE2 receptors cause upregulation of ACE2 receptors in hepatocytes leading to hepatocyte proliferation and resulting tissue injury [92]. (Image created with Biorender.com)

SARS-CoV-2 also adversely affects the digestive system through inflammation and viremia. Once the virus is bound to ACE2 receptor, ACE2 expression in hepatic tissue is upregulated, leading to compensatory proliferation of hepatocytes and resulting tissue injury [92] (Fig. 3). Studies have revealed presence of viral nucleic acid in stool samples of up to 53.4% of patients [93, 94]. The virus may also cause digestive symptoms by disrupting the intestinal microbiota which play a variety of significant physiological roles in the body, including maturation of the body's immune system, and antibacterial effects [95]. Alterations in the composition and function of the digestive tract microbiota can affect the respiratory tract due to common mucosal immune system, and vice versa. This “gut-lung axis” effect [96, 97] may be the reason why patients with COVID-19 pneumonia often also experience digestive symptoms.

### Musculoskeletal manifestations

Starting from the onset of symptoms to advanced stages of the COVID-19 disease, musculoskeletal symptoms, including myalgia, arthralgia, and fatigue are almost always present [98], although they do not seem to be associated with disease severity [30]. Musculoskeletal symptoms studied in this review are summarized in Table 1. Fatigue, recorded in 66.9% (49.9–73.6) of the patients was the most frequently experienced symptom.

#### Bridge to Rheumatology

A study from Korea observed an association between a rise in the incidence of Rheumatoid Arthritis (RA) and endemic human coronavirus, parainfluenza virus, and metapneumovirus infections [99], raising the concern that COVID-19 pandemic could potentially follow suit. However, currently no research suggests development of autoimmune inflammatory arthritis, such as RA because of infection with SARS-CoV-2 or any of the other known human coronaviruses.

**Fig. 4:**
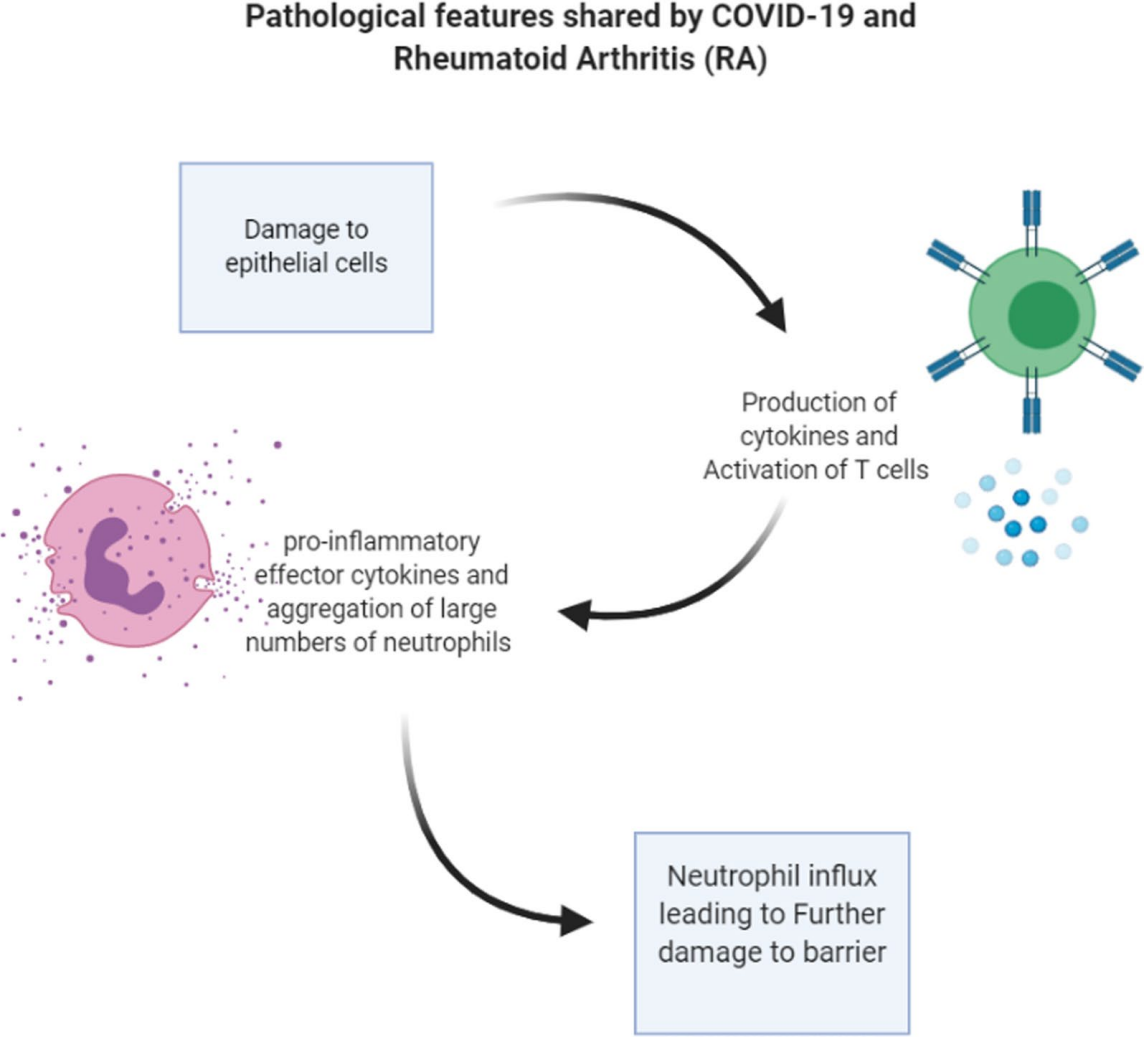
Pathological features shared by COVID-19 and RA: In both diseases there is immense inflammation of structures that form the inner surfaces of the body, and tissue damage and responses that eventually lead to organ failure. In COVID-19, alveolar epithelial cell damage and T cell activation that cause an increase in local production of pro-inflammatory effector cytokines and exaggerated accumulation of neutrophils and macrophages leading to a profound and uncontrolled immune response. Barrier damage, activation of T-cells, production of effector cytokines and neutrophil influx are also pertinent to synovitis, and RA shares some of these mediators with COVID-19. In COVID-19, structural damage and inflammation in COVID-19 largely confined to the lungs, which are destroyed progressively. Furthermore, and most likely due to activation of robust interleukin-6, COVID-19 mounts systemic acute-phase responses, similar to those in RA [81]. (Image created with Biorender.com)

The mode of action followed by pro-inflammatory effector cytokines in the alveolar membranes during severe COVID-19 is to some extent similar to that of primary cytokines targeted during treatment of RA [81] (Fig. 4). Both diseases cause significant inflammation of structures present on inner surfaces of the body and can trigger tissue damage and responses that result in organ failure. COVID-19 comprises extensive or uncontrolled host immune response, manifested as alveolar epithelial cells damage and T-cell activation in the lungs. The result is an increase in local production of pro-inflammatory effector cytokines and exaggerated accumulation of neutrophils and macrophages. [81]. Barrier damage, activation of T-cells, production of effector cytokines and influx of neutrophils are also main features of synovitis, and some mediators are common to both COVID-19 and RA. Furthermore, most likely due to robust activation of interleukin-6 the acute-phase systemic responses of COVID-19 and RA are similar. Cytokine inhibition, thus, may be a possible consideration for treatment of COVID-19, given the hyper-inflammatory state behind the pathology [81]. This may also affect treatment recommendations for RA. The risk of r contracting COVID-19 is not higher for people with arthritis, and anti-rheumatic treatment should not be stopped pre-emptively, during the pandemic [81].

### Dermatological manifestations

Of the studies reviewed, only one study reported non-specific “rash” in 0.18% of patients presenting with COVID-19 [30].

A study on dermatologic manifestations associated with COVID‐19 revealed that out of a total of 88 patients, 18 manifested skin involvement, but only 8 developed skin lesions at the onset of the disease [100]. A high percentage of the 8 individuals had erythematous rashes, while 3 developed widespread urticaria and chicken‐pox like vesicles [100]. A case of COVID‐19 with fever and morbilliform rash as the primary presenting sign [101] and another patient with a petechial skin rash as the only presenting sign were also reported [102]. Additionally, two further cases of COVID‐19 presenting with chilblain‐like lesions were also recorded [103].

Minimally invasive autopsies of COVID-19 patients have revealed destruction and necrosis of the parenchyma with formation of hyaline thrombi in small vessels in lung and other organs [104]. These findings could explain the underlying pathology of dermal manifestations and the causes of acral ischemia reported in patients with COVID‐19 in Italy [104]. Furthermore, cases have been shown to present initially with red–purple papules on hands and feet, which either evolved into hemorrhagic bullae or developed a blackish crust [105].

## Comorbidities

Cardiovascular diseases including but not limited to hypertension, diabetes, renal disease, and chronic obstructive pulmonary diseases (COPD) as a result of smoking are among the most frequently reported underlying comorbidities in hospitalized patients with COVID-19 [106]. Table 2 summarizes the comorbidities recorded in this study. According to this review, hypertension [28.6% (10.8–45.4)] was the most common co-morbidity, followed by cardiovascular diseases (coronary artery disease, congestive heart failure, vasculitis) [14.6% (10.1–21.4)], and diabetes [13.2% (10.2–30.1)]. Chen et al. and Zhang et al. reported presence of cardiovascular and cerebrovascular diseases collectively in 40.4% and 14.3% of their participants respectively [72, 87].

**Table 2:** Summarizes the frequencies of comorbidities in terms of median and interquartile range (IQR) recorded for COVID-19 patients by studies included in this review

Comorbidity	Frequency (%)		Studies reported
	Median (%)	IQR	
Hypertension	28.60	10.8–45.4	8
Cardiovascular diseases	14.60	10.1–21.4	9
Diabetes	13.20	10.2–30.1	12
Cerebrovascular diseases	8.30	2.1–14.3	6
Chronic lung disease	3.20	1.4–15.9	4
Chronic kidney disease	7.20	2.9–20.8	7
Immunocompromised state	4.80	1.1–11.4	6
Chronic liver disease	2.70	1.8–5.0	6

Diabetes was the most frequently recorded comorbidity but hypertension had the highest prevalance amongst patients of the studies that recorded hypertension as a comorbididty. Obesity, underlying endocrine disorders, autoimmune disease, gastrointestinal diseases and obstructive sleep apnea were aslo noted

Chen et al. [73] and Zhang et al. [83] reported presence of cardiovascular and cerebrovascular diseases collectively in 40.4% and 14.3% of their participants respectively

Chen et al. [73] and Zhang et al. [83] also reported presence of underlying chronic gastrointestinal diseases in 11.1% and 9.3%, and underlying endocrine diseases other than diabetes—such as thyroid disorders—in 1.1% and 3.6% of the patients respectively

Richardson et al. [107] and Goyal et al. [108] also reported obesity in 60.6% and 35.8% of their participants

Chen et al. also recorded presence of autoimmune disease in 1.1% of their participants [73]

Obstructive Sleep Apnea (OSA) was noted by Richardson [106], Arentz [109], and Bhatraju [66] as comorbidity amongst 2.7%, 28.8% and 20.8% of their participants respectively

Chen et al. and Zhang et al. also reported presence of underlying chronic gastrointestinal diseases in 11.1% and 9.3% of the population, and underlying endocrine diseases other than diabetes, such as thyroid disorders in 1.1% and 3.6% of the population respectively [72, 105].

Richardson et al. [107] and Goyal et al. [108] reported obesity in 60.6% and 35.8% of their participants. Chen et al. recorded presence of autoimmune disease in 1.1% of their participants[72]. Obstructive Sleep Apnea (OSA) was noted by Richardson et al. [107], Arentz et.al [109], and Bhatraju et al. [66] as a comorbidity amongst 2.7%, 28.8% and 20.8% of their participants respectively. The studies revealed no significant association of obesity with disease severity. Of the studies that recorded the smoking status of COVID-19 patients, 8.1% (5.4–18.1) smoked or had a history of smoking.

### The link between comorbid conditions and COVID-19

Since several features of chronic diseases are similar to those of infectious disorders for example the pro-inflammatory state and weakened innate immune response, their pathogeneses may be linked to COVID-19. Diabetes results partly from release of inflammatory mediators, especially interleukin-1β and TNFα, caused by aggregation of activated innate immune cells in metabolic tissues, thereby causing β-cell damage and systemic insulin resistance [110]. Metabolic disorders can also impair immune function by disrupting function of macrophage and lymphocyte [111] and through increasing the susceptibility to disease complications [112]. The levels of different inflammatory markers in the mortality group have understandably been considered to be higher than those in the survival group because the absolute count levels of CD3^+^ T cells, CD3^+^CD8^+^ T cells, and CD3^+^CD4^+^ T cells in the mortality group have been found to be significantly lower than those in the survival group [113]. Spinetti et al. found immune response dysfunction, manifested as reduces mHLA-DR expression on circulating CD14 + monocytes, was more common among ICU patients with severe COVID-19 illness when compared to non-critically ill hospitalized COVID-19 patients [17]. Patients with diabetes or hypertension have higher plasma plasminogen levels [114]. It is hypothesized that plasminogen may increase the ability of SARS-CoV-2 to bind to ACE2 receptors and aid its entry into host cells [115]. Data also suggest increased ACE2 activity in patients with cardiovascular diseases and diabetes [116, 117]. Increased ACE2 in diabetic or hypertensive patients with ACE inhibitors (ACEIs) or angiotensin II type 1 receptor antagonists may promote SARS-CoV-2 infection [118]. While studying seasonal influenza in a prospective case–control study, Hong et al.[119] established that diabetes and chronic cardiovascular disease had a significant association with development of disease-associated complications, and that diabetes independently increased the risk for severe seasonal influenza (OR 3.63, 95% CI 1.15–11.51, *p* = 0.02). Another study revealed that risk of mortality for patients with severe influenza was higher in those who had COPD (OR 1.49, 95% CI 1.10–2.01), and cardiovascular disease (OR 2.92, 95% CI 1.76–4.86) or hypertension (OR 1.49, 95% CI 1.10–2.10) [120].

A meta-analysis concluded that a history of Chronic Kidney Disease (CKD) in COVID-19 patients may increase the risk of disease severity [121]. Another study found that amongst patients with COVID-19 and underlying CKD, 83.93% of cases had severe disease with 53.33% mortality, while amongst those with liver diseases, 57.33% were severe with a mortality of 17. 65% [122].

Studies have shown obese COVID-19 patients to have a 37% higher rate of in-hospital death [123] and higher risk of contracting severe pneumonia [123] compared to non-obese patients. Smoking and older age group patients have a higher number of ACE2 receptors [15], which may explain why, overall, that severe cases belong to the older age group [31] with significantly more comorbidities than non-severe patients. Thus, as suggested by these results, age and comorbidities are risk factors for critical stage of the disease [9].

## COVID-19 and children

On January 20th 2020, the first case of SARS-COV-2 infection in pediatric age group was confirmed in Shenzhen, China [124]. Earlier in the course of the outbreak, SARS-CoV-2 infection was more relevant amongst adults and individual equal to or older than 15 years of age [125]. Early data from Chinese Centers for Diseases Control and Prevention reported that 0.9% of the total confirmed cases comprised children aged 0–10 years and 1.2% were individuals aged 10–19 years [126]. However, with the increase in the number infected adult contacts and more disease screening, the number infected children and young adults also increased, mainly with mild infections [127]. This increase in prevalence of SAR-CoV-2 infection in younger age group following the initial course of the pandemic is somewhat similar to the increased prevalence of disease cases observed in younger age groups following the initial phases of the pandemic during in the 1918 Spanish Flu [128] and 2009 H1N1 influenza outbreak [129]. Currently, the virus is believed to infect both children and adults at a similar rate[130], however the disease in children is milder in an overwhelming majority of cases [131].

Clinical characteristics of children with COVID‐19 can be asymptomatic infection, mild, common, severe, and critically severe [132]. Most children diagnosed with the disease experience mild symptoms, faster recovery, shorter detoxification time, and good prognosis [133]. Most children with COVID-19 have been found to present with fever and with some cases also having fatigue, myalgia, nasal congestion, runny nose, sneezing, sore throat, headache, dizziness, vomiting, and abdominal pain[125]. Children with COVID‐19 are believed to experience characteristics of “family‐aggregated” infections with a longer incubation period than adults[125] i.e., the mean incubation period for children is ∼6.5 days, which is longer than 5.2 days for adults. Nasal and pharyngeal detoxification in children occurs in about 6–21 days (mean = 12 days)[134]. A study from China found that 68% of children with COVID-19 had contact with an adult with SARS-CoV-2 infection. 90% of the participants were living closely with families [135].

While most children with COVID-19 experience a mild or asymptomatic infection, a rare complication—**Multisystem Inflammatory Syndrome in Children** (MIS-C)—has been associated with COVID-19 in children [136]. It manifests 4–6 weeks after infection as high-grade fever, organ dysfunction, and markedly raised inflammatory markers. Though unclear, the underlying pathology appears to have features of Kawasaki Disease (KD) and autoimmunity Although it has features similar to that of KD such as symptoms and organ dysfunction resulting from a hyperinflammatory response to an infections, T cell subgroups, interleukin (IL)-17A and presence of 1 l-6 in MIS-C in place of il-1 in KD, and arterial damage biomarkers set the two apart. Involvement of multiple antibodies in the pathogeneses of MIS-C has been suggested. A study from New York found that all of fifteen individuals in the study (age < 21 years) with MIS-C had fever at presentation [137]. Eighty-seven percent of the participants had gasterointestinal symptoms (vomiting, abdominal pain, and diarrhea). Three patients had cough or dyspnea, and two experienced chest pain. Almost half of the individuals had features suggestive of KD: rash in 47%, conjunctivitis in 27%, and swollen hands and feet in 27% of the cases.

## Current management options

### Management of COVID-19

Presently, no drugs have been approved by the FDA for pre and post-exposure prophylaxis. Disease prevention and infection control therefore remain the major components of COVID-19 management and vary considerably across different parts of the world depending on local disease prevalence, resources, socioeconomic conditions and economy. Detailed recommendations for infection control and personal protection for both general public and healthcare workers can be viewed in detail at the Centers for Disease Control and Prevention (CDC) website: https://www.cdc.gov/coronavirus/2019-ncov/index.html

### Treatment

Although as mentioned above, no drugs for COVID-19 treatment have been approved, a variety of drugs and investigational agents (summarized below and details of which can be viewed here: https://www.cdc.gov/coronavirus/2019-ncov/hcp/clinical-guidance-management-patients.html), are being assessed for the treatment of COVID-19 in multiple clinical trials around the world.

#### Remdesivir

Remdesivir is an intravenous (IV) investigational adenosine analog nucleotide pro-drug which has shown action against SARS-CoV-2 in vitro [138]. CDC recommends its use for the management of SARS-CoV-2 infection in hospitalized patients who at high risk of disease progression of receiving supplemental oxygen.

#### Other antiviral drugs

Chloroquine/Hydroxychloroquine are recommended for use in clinical trials but not for treatment of COVID-19. The combination of **hydroxychloroquine and azithromycin** is not recommended due to possible toxicities. HIV protease inhibitors, including **Lopinavir/ritonavir (AI)**, apart from disadvantageous pharmacodynamics, have not have not shown any therapeutic benefits against COVID-19 during clinical trials.

## Immune-based therapy

Data favoring the use of convalescent plasma for management of COVID-19 is not sufficient as of writing [139, 140]. Plasma obtained from individuals who have recovered from COVID-19 contains SARS-CoV-2 antibodies, [139], and can serve to form a plasma-derived concentrated antibody preparation known as the SARS-CoV-2 immune globulins. Both products may repress viral activity and inflammatory response. Additionally, neutralizing monoclonal bodies against SARS-CoV infection are under investigation in clinical trials.

Currently, the CDC does not have sufficient data to recommend either for or against the use of the recombinant human interleukin-1 receptor antagonist, Anakinra, or interferon-beta (for early disease in mild to moderate disease cases) for the treatment of COVID-19. The CDC panel, however, has recommended against the use of Anti-Intreleukin-6 receptor monoclonal antibodies (sarilumab, tocilizumab); or anti-Interleukin-6 monoclonal antibody (siltuximab), interferons alfa or beta (for severely or critically ill patients); and Janus kinase inhibitors (baricitinib, ruxolitinib, tofacitinib), and Bruton’s tyrosine kinase inhibitors (acalabrutinib, ibrutinib, zanubrutinib) in COVID-19 patients.

### Corticosteroids

Due to their strong anti-inflammatory properties, corticosteroids may prevent or alleviate systemic inflammatory responses in COVID-19 which may otherwise cause lung injury and multisystem organ dysfunction. Studies have reported both beneficial [141, 142] and harmful [143, 144] effects upon evaluating short courses of corticosteroids in patients with COVID-19.

### Dexamethasone

The data from a large, randomized, multicenter, open-label ongoing trial for hospitalized COVID-19 patients in the United Kingdom, known as the RECOVERY trial, have suggested that patients who received dexamethasone as a part of management had a reduced mortality rate when compared to those who received standard of care without dexamethasone [145]. CDC recommends use of dexamethasone for treatment of COVID-19 cases with severe disease where patients are hospitalized and require supplemental oxygen, oxygen through high-flow device or extracorporeal membrane oxygenation (ECMO).

Pharmacologic therapy of COVID-19 based on disease severity can be viewed here: https://www.covid19treatmentguidelines.nih.gov/therapeutic-management/

### Vaccine

As of writing, 89 vaccine candidates are enrolled in clinical trials involving humans, and at least 86 preclinical vaccines are under investigation on animals. A total of 26 vaccine candidates have reached phase 3 of clinical trials, 5 have been approved for limited use 8 approved for full (and emergency) use in some parts of the world, including the cavvines by Comirnaty (Pfizer-biontech), mRNA-1273 (Moderna), Vaxzevria (AstraZeneca), Convidecia (CanSinoBio), EpiVacCorona (BEKTOP), BBIBP-CorV (Sinopharm) CoronaVac (Sinovac), and Sinopharm-Wuhan. Updated information regarding COVID-19 vaccines can be found here: https://www.nytimes.com/interactive/2020/science/coronavirus-vaccine-tracker.html

## Conclusions

This review comprehensively discusses and illustrates the underlying pathological mechanisms that orchestrate COVID-19 disease behavior and the huge spectrum of its clinical manifestations. It compiles and brings valuable pieces of information published till data to one table and links them with one another. Treatment and management options available till date have also been briefly discussed. While a number of vaccine candidates have shown promising results, factors such as cost and logistics will in part determine the magnitude of importance of vaccines in putting an end to the pandemic. Thus, disease prevention and infection control remain the cornerstones of management of the disease.

Our study shows that while certain signs and symptoms are often associated present in multiple ways which are not otherwise typical of the disease. This may suggest that COVID-19 can also present as a multisystem disease. The high prevalence of comorbidities should indicate increased severity of the disease, high risk of deterioration, prolonged hospital stay and associated mortality in a giving population. In a clinical and surgical setting, absence for pathognomonic symptoms in a patient should not be considered synonymous to absence COVID-19.Based on our understanding, SARS-CoV-2 infection in the nervous system may extend to and affect the neural retina. A prospective study including autopsy biopsies may be conducted.

## Data Availability

The data explored during the current study was available from the corresponding author on reasonable request.

## Abbreviations

WHO: World Health Organization
ARDS: Acute respiratory distress syndrome
ICU: Intensive care unit
CNS: Central nervous system
CRS: Cytokine release syndrome
CRP: C-reactive protein
LDH: Lactate dehydrogenase
HAS2: HA-synthase-2
COPD: Chronic obstructive pulmonary diseases
ECMO: Extracorporeal membrane oxygenation

## References

[1] Wu Y-C, Chen C-S, Chan Y-J (2020). The outbreak of COVID-19: An overview. J Chin Med Assoc.

[2] Cucinotta D, Vanelli M (2020). WHO declares COVID-19 a pandemic. Acta bio-medica: Atenei Parmensis.

[3] Jean S-S, Lee P-I, Hsueh P-R (2020). Treatment options for COVID-19: The reality and challenges. J Microbiol Immunol Infect.

[4] Felsenstein S, Herbert JA, McNamara PS, Hedrich CM (2020). COVID-19: Immunology and treatment options. Clin Immunol.

[5] Wang C, Horby PW, Hayden FG, Gao GF (2020). A novel coronavirus outbreak of global health concern. Lancet.

[6] Li L-q (2020). Huang T, Wang Y-q, Wang Z-p, Liang Y, Huang T-b, Zhang H-y, Sun W, Wang Y: COVID-19 patients' clinical characteristics, discharge rate, and fatality rate of meta-analysis. J Med Virol.

[7] Baqui P, Bica I, Marra V, Ercole A, van der Schaar M. Ethnic and regional variations in hospital mortality from COVID-19 in Brazil: a cross-sectional observational study. Lancet Glob Health 2020. 10.1016/S2214-109X(20)30285-0 PMC7332269 32622400

[8] Li B, Yang J, Zhao F, Zhi L, Wang X, Liu L, Bi Z, Zhao Y (2020). Prevalence and impact of cardiovascular metabolic diseases on COVID-19 in China. Clin Res Cardiol.

[9] Yang J, Zheng Y, Gou X, Pu K, Chen Z, Guo Q, Ji R, Wang H, Wang Y, Zhou Y (2020). Prevalence of comorbidities and its effects in patients infected with SARS-CoV-2: a systematic review and meta-analysis. Int J Infect Dis.

[10] Li Q, Guan X, Wu P, Wang X, Zhou L, Tong Y, Ren R, Leung KSM, Lau EHY, Wong JY (2020). Early transmission dynamics in Wuhan, China, of novel coronavirus-infected pneumonia. N Engl J Med.

[11] Wang W, Tang J, Wei F (2020). Updated understanding of the outbreak of 2019 novel coronavirus (2019-nCoV) in Wuhan, **China**. J Med Virol.

[12] Chen G, Wu D, Guo W, Cao Y, Huang D, Wang H, Wang T, Zhang X, Chen H, Yu H (2020). Clinical and immunological features of severe and moderate coronavirus disease 2019. J Clin Investig.

[13] Huang C, Wang Y, Li X, Ren L, Zhao J, Hu Y, Zhang L, Fan G, Xu J, Gu X (2020). Clinical features of patients infected with 2019 novel coronavirus in Wuhan, **China**. Lancet.

[14] Ren L-L, Wang Y-M, Wu Z-Q, Xiang Z-C, Guo L, Xu T, Jiang Y-Z, Xiong Y, Li Y-J, Li X-W (2020). Identification of a novel coronavirus causing severe pneumonia in human: a descriptive study. Chin Med J (Engl).

[15] Kakodkar P, Kaka N, Baig MN (2020). A comprehensive literature review on the clinical presentation, and management of the pandemic Coronavirus Disease 2019 (COVID-19). Cureus.

[16] Takahashi T, Wong P, Ellingson M, Lucas C, Klein J, Israelow B, Silva J, Oh J, Mao T, Tokuyama M et al. Sex differences in immune responses to SARS-CoV-2 that underlie disease outcomes. medRxiv 2020:2020.2006.2006.20123414.

[17] Spinetti T, Hirzel C, Fux M, Walti LN, Schober P, Stueber F, Luedi MM, Schefold JC. Reduced monocytic HLA-DR expression indicates immunosuppression in critically ill COVID-19 patients. Anesthesia & Analgesia 9000, Publish Ahead of Print. 10.1213/ANE.0000000000005044 PMC7288784 32925314

[18] Shi Y, Wang Y, Shao C, Huang J, Gan J, Huang X, Bucci E, Piacentini M, Ippolito G, Melino G (2020). COVID-19 infection: the perspectives on immune responses. Cell Death Differ.

[19] Benetti E, Tita R, Spiga O, Ciolfi A, Birolo G, Bruselles A, Doddato G, Giliberti A, Marconi C, Musacchia F et al: ACE2 gene variants may underlie interindividual variability and susceptibility to COVID-19 in the Italian population. medRxiv 2020:2020.2004.2003.20047977. 10.1038/s41431-020-0691-z PMC7366459 32681121

[20] Zhao J, Yang Y, Huang H, Li D, Gu D, Lu X, Zhang Z, Liu L, Liu T, Liu Y et al. Relationship between the ABO blood group and the coronavirus disease 2019 (COVID-19) susceptibility. Clin Infect Diseases 2020. 10.1093/cid/ciaa1150 PMC7454371 32750119

[21] Ellinghaus D, Degenhardt F, Bujanda L, Buti M, Albillos A, Invernizzi P, Fernández J, Prati D, Baselli G, Asselta R et al. Genomewide Association Study of Severe Covid-19 with respiratory failure. New Engl J Med. 2020. 10.1056/NEJMoa2020283 PMC7315890 32558485

[22] Miller A, Reandelar MJ, Fasciglione K, Roumenova V, Li Y, Otazu GH: Correlation between universal BCG vaccination policy and reduced morbidity and mortality for COVID-19: an epidemiological study. medRxiv 2020:2020.2003.2024.20042937.

[23] Ma Y, Zhao Y, Liu J, He X, Wang B, Fu S, Yan J, Niu J, Luo B: Effects of temperature variation and humidity on the mortality of COVID-19 in Wuhan. medRxiv 2020:2020.2003.2015.20036426. 10.1016/j.scitotenv.2020.138226 PMC7142681 32408453

[24] Haynes N, Cooper LA, Albert MA (2020). Cardiologists AoB: at the heart of the matter: unmasking and addressing the toll of COVID-19 on diverse populations. Circulation.

[25] Mao L, Jin H, Wang M, Hu Y, Chen S, He Q, Chang J, Hong C, Zhou Y, Wang D (2020). Neurologic manifestations of hospitalized patients with coronavirus disease 2019 in Wuhan, China. JAMA Neurol.

[26] Helms J, Kremer S, Merdji H, Clere-Jehl R, Schenck M, Kummerlen C, Collange O, Boulay C, Fafi-Kremer S, Ohana M (2020). Neurologic features in severe SARS-CoV-2 infection. N Engl J Med.

[27] Lechien JR, Chiesa-Estomba CM, De Siati DR, Horoi M, Le Bon SD, Rodriguez A, Dequanter D, Blecic S, El Afia F, Distinguin L (2020). Olfactory and gustatory dysfunctions as a clinical presentation of mild-to-moderate forms of the coronavirus disease (COVID-19): a multicenter European study. Eur Arch Oto-Rhino-Laryngol.

[28] Gutiérrez-Ortiz C, Méndez A, Rodrigo-Rey S, San Pedro-Murillo E, Bermejo-Guerrero L, Gordo-Mañas R, de Aragón-Gómez F, Benito-León J (2020). Miller Fisher Syndrome and polyneuritis cranialis in COVID-19. Neurology.

[29] Toscano G, Palmerini F, Ravaglia S, Ruiz L, Invernizzi P, Cuzzoni MG, Franciotta D, Baldanti F, Daturi R, Postorino P (2020). Guillain-Barré Syndrome Associated with SARS-CoV-2. N Engl J Med.

[30] Guan W-j, Ni Z-y, Hu Y, Liang W-h, Ou C-q, He J-x, Liu L, Shan H, Lei C-l, Hui DSC et al. Clinical characteristics of Coronavirus Disease 2019 in China. New Engl J Med. 2020;382(18), 1708–1720. 10.1056/NEJMoa2002032 PMC7092819 32109013

[31] Wang D, Hu B, Hu C, Zhu F, Liu X, Zhang J, Wang B, Xiang H, Cheng Z, Xiong Y (2020). Clinical characteristics of 138 hospitalized patients with 2019 novel coronavirus-infected pneumonia in Wuhan, **China**. JAMA.

[32] Oxley TJ, Mocco J, Majidi S, Kellner CP, Shoirah H, Singh IP, De Leacy RA, Shigematsu T, Ladner TR, Yaeger KA (2020). Large-vessel stroke as a presenting feature of Covid-19 in the young. N Engl J Med.

[33] Avula A, Nalleballe K, Narula N, Sapozhnikov S, Dandu V, Toom S, Glaser A, Elsayegh D (2020). COVID-19 presenting as stroke. Brain Behav Immun.

[34] Kalfaoglu B, Almeida-Santos J, Adele Tye C, Satou Y, Ono M. T-cell hyperactivation and paralysis in severe COVID-19 infection revealed by single-cell analysis. bioRxiv 2020:2020.2005.2026.115923. 10.3389/fimmu.2020.589380 PMC7596772 33178221

[35] Wang Y, Wang Y, Chen Y, Qin Q (2020). Unique epidemiological and clinical features of the emerging 2019 novel coronavirus pneumonia (COVID-19) implicate special control measures. J Med Virol.

[36] Heneka MT, Golenbock D, Latz E, Morgan D, Brown R (2020). Immediate and long-term consequences of COVID-19 infections for the development of neurological disease. Alzheimer's Res Therapy.

[37] Tsai L, Hsieh S, Chang Y (2005). Neurological manifestations in severe acute respiratory syndrome. Acta Neurol Taiwan.

[38] Wu A, Peng Y, Huang B, Ding X, Wang X, Niu P, Meng J, Zhu Z, Zhang Z, Wang J (2020). Genome composition and divergence of the novel coronavirus (2019-nCoV) originating in China. Cell Host Microbe.

[39] Huang Y, Yang C (2020). Xu X-f, Xu W, Liu S-w: Structural and functional properties of SARS-CoV-2 spike protein: potential antivirus drug development for COVID-19. Acta Pharmacol Sin.

[40] Heneka MT, Kummer MP, Stutz A, Delekate A, Schwartz S, Vieira-Saecker A, Griep A, Axt D, Remus A, Tzeng T-C (2013). NLRP3 is activated in Alzheimer’s disease and contributes to pathology in APP/PS1 mice. Nature.

[41] Feng Z, Qi S, Zhang Y, Qi Z, Yan L, Zhou J, He F, Li Q, Yang Y, Chen Q (2017). Ly6G+ neutrophil-derived miR-223 inhibits the NLRP3 inflammasome in mitochondrial DAMP-induced acute lung injury. Cell Death Dis.

[42] Siu KL, Yuen KS, Castano-Rodriguez C, Ye ZW, Yeung ML, Fung SY, Yuan S, Chan CP, Yuen KY, Enjuanes L (2019). Severe acute respiratory syndrome Coronavirus ORF3a protein activates the NLRP3 inflammasome by promoting TRAF3-dependent ubiquitination of ASC. FASEB J.

[43] Ding H-G, Deng Y-Y (2018). Yang R-q, Wang Q-S, Jiang W-Q, Han Y-L, Huang L-Q, Wen M-Y, Zhong W-H, Li X-S: Hypercapnia induces IL-1β overproduction via activation of NLRP3 inflammasome: implication in cognitive impairment in hypoxemic adult rats. J Neuroinflammation.

[44] Tejera D, Mercan D, Sanchez-Caro JM, Hanan M, Greenberg D, Soreq H, Latz E, Golenbock D, Heneka MT (2019). Systemic inflammation impairs microglial Aβ clearance through NLRP 3 inflammasome. EMBO J.

[45] Venegas C, Kumar S, Franklin BS, Dierkes T, Brinkschulte R, Tejera D, Vieira-Saecker A, Schwartz S, Santarelli F, Kummer MP (2017). Microglia-derived ASC specks cross-seed amyloid-β in Alzheimer’s disease. Nature.

[46] Ising C, Venegas C, Zhang S, Scheiblich H, Schmidt SV, Vieira-Saecker A, Schwartz S, Albasset S, McManus RM, Tejera D (2019). NLRP3 inflammasome activation drives tau pathology. Nature.

[47] Iwashyna TJ, Ely EW, Smith DM, Langa KM (2010). Long-term cognitive impairment and functional disability among survivors of severe sepsis. JAMA.

[48] Widmann CN, Heneka MT (2014). Long-term cerebral consequences of sepsis. Lancet Neurol.

[49] Rodriguez-Morales AJ, Cardona-Ospina JA, Gutiérrez-Ocampo E, Villamizar-Peña R, Holguin-Rivera Y, Escalera-Antezana JP, Alvarado-Arnez LE, Bonilla-Aldana DK, Franco-Paredes C, Henao-Martinez AF (2020). Clinical, laboratory and imaging features of COVID-19: a systematic review and meta-analysis. Travel Med Infect Dis.

[50] Girard TD, Thompson JL, Pandharipande PP, Brummel NE, Jackson JC, Patel MB, Hughes CG, Chandrasekhar R, Pun BT, Boehm LM (2018). Clinical phenotypes of delirium during critical illness and severity of subsequent long-term cognitive impairment: a prospective cohort study. Lancet Respir Med.

[51] Sasannejad C, Ely EW, Lahiri S (2019). Long-term cognitive impairment after acute respiratory distress syndrome: a review of clinical impact and pathophysiological mechanisms. Crit Care.

[52] Zhou L, Zhang M, Wang J, Gao J (2020). Sars-Cov-2: Underestimated damage to nervous system. Travel Med Infect Dis.

[53] Cao W, Fang Z, Hou G, Han M, Xu X, Dong J, Zheng J (2020). The psychological impact of the COVID-19 epidemic on college students in China. Psychiatry Res.

[54] Banerjee D (2020). The COVID-19 outbreak: Crucial role the psychiatrists can play. Asian J Psychiatry.

[55] Ahmed MZ, Ahmed O, Aibao Z, Hanbin S, Siyu L, Ahmad A (2020). Epidemic of COVID-19 in China and associated psychological problems. Asian J Psychiatry.

[56] Wu PE, Styra R, Gold WL (2020). Mitigating the psychological effects of COVID-19 on health care workers. CMAJ.

[57] Gangaputra SS, Patel SN. Ocular symptoms among nonhospitalized patients who underwent COVID-19 testing. Ophthalmology. 10.1016/j.ophtha.2020.06.037 PMC7308009 32585259

[58] Wu P, Duan F, Luo C, Liu Q, Qu X, Liang L, Wu K (2020). Characteristics of Ocular Findings of Patients With Coronavirus Disease 2019 (COVID-19) in Hubei Province, **China**. JAMA Ophthalmol.

[59] Cheema M, Aghazadeh H, Nazarali S, Ting A, Hodges J, McFarlane A, Kanji JN, Zelyas N, Damji KF, Solarte C (2020). Keratoconjunctivitis as the initial medical presentation of the novel coronavirus disease 2019 (COVID-19). Can J Ophthalmol.

[60] Wu P, Liang L, Chen C, Nie S: A child confirmed COVID-19 with only symptoms of conjunctivitis and eyelid dermatitis. Graefe's Arch Clin Exp Ophthalmol. 2020:1. 10.1007/s00417-020-04708-6 PMC7181963 32333104

[61] Li X, Wang M, Dai J, Wang W, Yang Y, Jin W: Novel coronavirus disease with conjunctivitis and conjunctivitis as first symptom: two cases report. Chin J Exp Ophthalmol. 2020; 38(4).

[62] Casalino G, Monaco G, Di Sarro PP, David A, Scialdone A: Coronavirus disease,  (2019). presenting with conjunctivitis as the first symptom. Eye.

[63] Scalinci SZ, Battagliola ET. Conjunctivitis can be the only presenting sign and symptom of COVID-19. IDCases 2020:e00774. 10.1016/j.idcr.2020.e00774 PMC7195291 32373467

[64] Olivia Li J-P, Shantha J, Wong TY, Wong EY, Mehta J, Lin H, Lin X, Strouthidis NG, Park KH, Fung AT (2020). Preparedness among ophthalmologists: during and beyond the COVID-19 pandemic. Ophthalmology.

[65] Lovato A, de Filippis C. Clinical presentation of COVID-19: A systematic review focusing on upper airway symptoms. Ear Nose Throat J 2020:0145561320920762. 10.1177/0145561320920762 32283980

[66] Bhatraju PK, Ghassemieh BJ, Nichols M, Kim R, Jerome KR, Nalla AK, Greninger AL, Pipavath S, Wurfel MM, Evans L (2020). Covid-19 in critically Ill patients in the Seattle Region—Case series. N Engl J Med.

[67] Gane SB, Kelly C, Hopkins C (2020). Isolated sudden onset anosmia in COVID-19 infection. A novel syndrome. Rhinology.

[68] Hopkins C, Surda P, Kumar N (2020). Presentation of new onset anosmia during the COVID-19 pandemic. Rhinology.

[69] Vaira LA, Salzano G, Deiana G, De Riu G (2020). Anosmia and ageusia: common findings in COVID-19 patients. Laryngoscope.

[70] Suzuki M, Saito K, Min WP, Vladau C, Toida K, Itoh H, Murakami S (2007). Identification of viruses in patients with postviral olfactory dysfunction. Laryngoscope.

[71] Baig AM, Khaleeq A, Ali U, Syeda H (2020). Evidence of the COVID-19 virus targeting the CNS: tissue distribution, host–virus interaction, and proposed neurotropic mechanisms. ACS Chem Neurosci.

[72] Zhou F, Yu T, Du R, Fan G, Liu Y, Liu Z, Xiang J, Wang Y, Song B, Gu X (2020). Clinical course and risk factors for mortality of adult inpatients with COVID-19 in Wuhan, China: a retrospective cohort study. Lancet.

[73] Chen N, Zhou M, Dong X, Qu J, Gong F, Han Y, Qiu Y, Wang J, Liu Y, Wei Y (2020). Epidemiological and clinical characteristics of 99 cases of 2019 novel coronavirus pneumonia in Wuhan, China: a descriptive study. Lancet.

[74] Han C, Duan C, Zhang S, Spiegel B, Shi H, Wang W, Zhang L, Lin R, Liu J, Ding Z (2020). Digestive Symptoms in COVID-19 Patients With Mild Disease Severity: Clinical Presentation, Stool Viral RNA Testing, and Outcomes. Am J Gastroenterol.

[75] Liu K, Fang Y-Y, Deng Y, Liu W, Wang M-F, Ma J-P, Xiao W, Wang Y-N, Zhong M-H, Li C-H (2020). Clinical characteristics of novel coronavirus cases in tertiary hospitals in Hubei Province. Chin Med J (Engl).

[76] Tian S, Xiong Y, Liu H, Niu L, Guo J, Liao M, Xiao S-Y: Pathological study of the (2019). novel coronavirus disease (COVID-19) through postmortem core biopsies. Mod Pathol.

[77] Barton LM, Duval EJ, Stroberg E, Ghosh S, Mukhopadhyay S (2020). Covid-19 autopsies, oklahoma, usa. Am J Clin Pathol.

[78] Bell TJ, Brand OJ, Morgan DJ, Salek-Ardakani S, Jagger C, Fujimori T, Cholewa L, Tilakaratna V, Östling J, Thomas M (2019). Defective lung function following influenza virus is due to prolonged, reversible hyaluronan synthesis. Matrix Biol.

[79] Xu Z, Shi L, Wang Y, Zhang J, Huang L, Zhang C, Liu S, Zhao P, Liu H, Zhu L (2020). Pathological findings of COVID-19 associated with acute respiratory distress syndrome. Lancet Respir Med.

[80] Hallgren R, Samuelsson T, Laurent TC, Modig J (1989). Accumulation of hyaluronan (hyaluronic acid) in the lung in adult respiratory distress syndrome. Am Rev Respir Dis.

[81] Schett G, Manger B, Simon D, Caporali R (2020). COVID-19 revisiting inflammatory pathways of arthritis. Nat Rev Rheumatol.

[82] Shi S, Qin M, Shen B, Cai Y, Liu T, Yang F, Gong W, Liu X, Liang J, Zhao Q et al. Association of cardiac injury with mortality in hospitalized patients with COVID-19 in Wuhan, China. JAMA Cardiol. 2020. 10.1001/jamacardio.2020.0950 PMC7097841 32211816

[83] Zhang J-j, Dong X, Cao Y-y, Yuan Y-d, Yang Y-b, Yan Y-q, Akdis CA, Gao Y-d. Clinical characteristics of 140 patients infected with SARS-CoV-2 in Wuhan, China. Allergy. 10.1111/all.14238 32077115

[84] Guo T, Fan Y, Chen M, Wu X, Zhang L, He T, Wang H, Wan J, Wang X, Lu Z (2020). Cardiovascular implications of fatal outcomes of patients with coronavirus disease 2019 (COVID-19). JAMA Cardiol.

[85] Ruan Q, Yang K, Wang W, Jiang L, Song J (2020). Clinical predictors of mortality due to COVID-19 based on an analysis of data of 150 patients from Wuhan, **China**. Intensive Care Med.

[86] Fried JA, Ramasubbu K, Bhatt R, Topkara VK, Clerkin KJ, Horn E, Rabbani L, Brodie D, Jain SS, Kirtane A (1930). The variety of cardiovascular presentations of COVID-19. Circulation.

[87] Jones VG, Mills M, Suarez D, Hogan CA, Yeh D, Segal JB, Nguyen EL, Barsh GR, Maskatia S, Mathew R (2020). COVID-19 and Kawasaki disease: novel virus and novel case. Hosp Pediatr.

[88] Lippi G, Plebani M, Henry BM (2020). Thrombocytopenia is associated with severe coronavirus disease 2019 (COVID-19) infections: a meta-analysis. Clin Chim Acta.

[89] Panigada M, Bottino N, Tagliabue P, Grasselli G, Novembrino C, Chantarangkul V, Pesenti A, Peyvandi F, Tripodi A. Hypercoagulability of COVID-19 patients in intensive care unit: a report of thromboelastography findings and other parameters of hemostasis. J Thrombosis Haemostasis. 10.1111/jth.14850 PMC9906150 32302438

[90] Zhang J, Liu P, Wang M, Wang J, Chen J, Yuan W, Li M, Xie Z, Dong W, Li H (2020). The clinical data from 19 critically ill patients with coronavirus disease 2019: a single-centered, retrospective, observational study. J Public Health.

[91] Pan L, Mu M, Yang P, Sun Y, Wang R, Yan J, Li P, Hu B, Wang J, Hu C (2020). Clinical Characteristics of COVID-19 Patients With Digestive Symptoms in Hubei, China: A Descriptive, Cross-Sectional, Multicenter Study. Am J Gastroenterol.

[92] Guan GW, Gao L, Wang JW, Wen XJ, Mao TH, Peng SW, Zhang T, Chen XM, Lu FM (2020). Exploring the mechanism of liver enzyme abnormalities in patients with novel coronavirus-infected pneumonia. Chin J Hepatol.

[93] Tang A, Tong ZD, Wang HL, Dai YX, Li KF, Liu JN, Wu WJ, Yuan C, Yu ML, Li P (2020). Detection of novel coronavirus by RT-PCR in stool specimen from asymptomatic child, China. Emerg Infect Dis.

[94] Xiao F, Tang M, Zheng X, Liu Y, Li X, Shan H (2020). Evidence for gastrointestinal infection of SARS-CoV-2. Gastroenterology.

[95] Li M, Wang B, Zhang M, Rantalainen M, Wang S, Zhou H, Zhang Y, Shen J, Pang X, Zhang M (2008). Symbiotic gut microbes modulate human metabolic phenotypes. Proc Natl Acad Sci.

[96] He Y, Wen Q, Yao F, Xu D, Huang Y, Wang J (2017). Gut–lung axis: The microbial contributions and clinical implications. Crit Rev Microbiol.

[97] Budden KF, Gellatly SL, Wood DL, Cooper MA, Morrison M, Hugenholtz P, Hansbro PM (2017). Emerging pathogenic links between microbiota and the gut–lung axis. Nat Rev Microbiol.

[98] Cipollaro L, Giordano L, Padulo J, Oliva F, Maffulli N. Musculoskeletal symptoms in SARS-CoV-2 (COVID-19) patients. In*.*: BioMed Central; 2020. 10.1186/s13018-020-01702-w PMC7232908 32423471

[99] Joo YB, Lim Y-H, Kim K-J, Park K-S, Park Y-J (2019). Respiratory viral infections and the risk of rheumatoid arthritis. Arthritis Res Ther.

[100] Recalcati S: Cutaneous manifestations in COVID‐19: a first perspective. J Eur Acad Dermatol Venereol. 2020. 10.1111/jdv.16387 32215952

[101] Hunt M, Koziatek C. A case of COVID-19 pneumonia in a young male with full body rash as a presenting symptom. Clin Pract Cases Emerg Med. 2020. 10.5811/cpcem.2020.3.47349 PMC7220001 32282312

[102] Joob B, Wiwanitkit V (2020). COVID-19 can present with a rash and be mistaken for Dengue. J Am Acad Dermatol.

[103] Alramthan A, Aldaraji W (2020). Two cases of COVID-19 presenting with a clinical picture resembling chilblains: first report from the Middle East. Clin Exp Dermatol.

[104] Yao X, Li T, He Z, Ping Y, Liu H, Yu S, Mou H, Wang L, Zhang H, Fu W (2020). A pathological report of three COVID-19 cases by minimally invasive autopsies. Chin J Pathol.

[105] Mazzotta F, Troccoli T (2020). Acute acro-ischemia in the child at the time of COVID-19. Eur J Pediatr Dermatol.

[106] Emami A, Javanmardi F, Pirbonyeh N, Akbari A (2020). Prevalence of underlying diseases in hospitalized patients with COVID-19: a systematic review and meta-analysis. Arch Acad Emerg Med.

[107] Richardson S, Hirsch JS, Narasimhan M, Crawford JM, McGinn T, Davidson KW (2020). Consortium atNC-R: presenting characteristics, comorbidities, and outcomes among 5700 patients hospitalized with COVID-19 in the New York City Area. JAMA.

[108] Goyal P, Choi JJ, Pinheiro LC, Schenck EJ, Chen R, Jabri A, Satlin MJ, Campion TR, Nahid M, Ringel JB (2020). Clinical characteristics of Covid-19 in New York City. N Engl J Med.

[109] Arentz M, Yim E, Klaff L, Lokhandwala S, Riedo FX, Chong M, Lee M (2020). Characteristics and outcomes of 21 critically Ill patients with COVID-19 in Washington State. JAMA.

[110] Odegaard JI, Chawla A (2012). Connecting type 1 and type 2 diabetes through innate immunity. Cold Spring Harb Perspect Med.

[111] Dooley KE, Chaisson RE (2009). Tuberculosis and diabetes mellitus: convergence of two epidemics. Lancet Infect Dis.

[112] Badawi A, Ryoo SG (2016). Prevalence of comorbidities in the Middle East respiratory syndrome coronavirus (MERS-CoV): a systematic review and meta-analysis. Int J Infect Dis.

[113] Guo L, Wei D, Wu Y, Zhou M, Zhang X, Li Q, Qu J (2019). Clinical features predicting mortality risk in patients with viral pneumonia: the MuLBSTA score. Front Microbiol.

[114] Ray EC, Miller RG, Demko JE, Costacou T, Kinlough CL, Demko CL, Unruh ML, Orchard TJ, Kleyman TR (2018). Urinary plasmin(ogen) as a prognostic factor for hypertension. Kidney Int Rep.

[115] Ji H-L, Zhao R, Matalon S, Matthay MA (2020). Elevated Plasmin(ogen) as a Common Risk Factor for COVID-19 Susceptibility. Physiol Rev.

[116] Walters TE, Kalman JM, Patel SK, Mearns M, Velkoska E, Burrell LM (2017). Angiotensin converting enzyme 2 activity and human atrial fibrillation: increased plasma angiotensin converting enzyme 2 activity is associated with atrial fibrillation and more advanced left atrial structural remodelling. Ep Europace.

[117] Úri K, Fagyas M, Kertész A, Borbély A, Jenei C, Bene O, Csanádi Z, Paulus WJ, Édes I, Papp Z. Circulating ACE2 activity correlates with cardiovascular disease development. J Renin-Angiotensin-Aldosterone Syst. 2016;**17**(4), 1470320316668435. 10.1177/1470320316668435 PMC5843890 27965422

[118] Fang L, Karakiulakis G, Roth M (2020). Are patients with hypertension and diabetes mellitus at increased risk for COVID-19 infection?. Lancet Respir Med.

[119] Hong K-W, Cheong HJ, Choi WS, Lee J, Wie S-H, Baek JH, Kim HY, Jeong HW, Kim WJ (2014). Clinical courses and outcomes of hospitalized adult patients with seasonal influenza in Korea, 2011–2012: Hospital-based Influenza Morbidity & Mortality (HIMM) surveillance. J Infect Chemother.

[120] Mertz D, Kim TH, Johnstone J, Lam P-P, Kuster SP, Fadel SA, Tran D, Fernandez E, Bhatnagar N, Loeb M (2013). Populations at risk for severe or complicated influenza illness: systematic review and meta-analysis. BMJ.

[121] Henry BM, Lippi G. Chronic kidney disease is associated with severe coronavirus disease 2019 (COVID-19) infection. Int Urol Nephrol. 2020;1–2 10.1007/s11255-020-02451-9 PMC7103107 32222883

[122] Oyelade T, Alqahtani J, Canciani G (2020). Prognosis of COVID-19 in patients with liver and kidney diseases: an early systematic review and meta-analysis. Trop Med Infect Dis.

[123] Docherty AB, Harrison EM, Green CA, Hardwick HE, Pius R, Norman L, Holden KA, Read JM, Dondelinger F, Carson G et al. Features of 16,749 hospitalised UK patients with COVID-19 using the ISARIC WHO Clinical Characterisation Protocol. medRxiv 2020:2020.2004.2023.20076042. 10.1136/bmj.m1985 PMC7243036 32444460

[124] Chan JF-W, Yuan S, Kok K-H, To KK-W, Chu H, Yang J, Xing F, Liu J, Yip CC-Y, Poon RW-S. A familial cluster of pneumonia associated with the 2019 novel coronavirus indicating person-to-person transmission: a study of a family cluster. Lancet 2020;395(10223), 514–523. 10.1016/S0140-6736(20)30154-9 PMC7159286 31986261

[125] She J, Liu L, Liu W (2020). COVID-19 epidemic: disease characteristics in children. J Med Virol.

[126] Lu X, Zhang L, Du H, Zhang J, Li YY, Qu J, Zhang W, Wang Y, Bao S, Li Y (2020). SARS-CoV-2 infection in children. N Engl J Med.

[127] Zhang Y (2020). The epidemiological characteristics of an outbreak of 2019 novel Coronavirus diseases (COVID-19)—China, 2020. China CDC Week.

[128] Olson DR, Simonsen L, Edelson PJ, Morse SS (2005). Epidemiological evidence of an early wave of the 1918 influenza pandemic in New York City. Proc Natl Acad Sci.

[129] Chowell G, Bertozzi SM, Colchero MA, Lopez-Gatell H, Alpuche-Aranda C, Hernandez M, Miller MA (2009). Severe respiratory disease concurrent with the circulation of H1N1 influenza. N Engl J Med.

[130] Jones TC, Mühlemann B, Veith T, Biele G, Zuchowski M, Hoffmann J, Stein A, Edelmann A, Corman VM, Drosten C. An analysis of SARS-CoV-2 viral load by patient age. MedRxiv 2020.

[131] Brodin P: Why is COVID‐19 so mild in children? In: Wiley Online Library; 2020. 10.1111/apa.15271 32212348

[132] Yi J, Xiaoxia L, Runming J. Novel coronavirus infections: standard/protocol/guideline recommendations for the diagnosis, prevention and control of the 2019 novel coronavirus infection in children (the second edition). Chin J Appl Clin Pediatr. 2020;**35**(2), 143–150.

[133] She J, Liu L, Liu W: Συνολική αναφορά εργασιών με τα χαρακτηριστικά της επιδημίας COVID‐19 στα παιδιά. Από το Περιοδικό Ιατρική Ιολογία του Απρίλη 15 2020. J Med Virol. 2020.

[134] Cai J, Xu J, Lin D: A case series of children with 2019 novel coronavirus infection: clinical and epidemiological features [published online February 28, 2020]. Clin Infect Dis. 10.1093/cid/ciaa198 PMC7108143 32112072

[135] Wang D, Ju X, Xie F, Lu Y, Li F, Huang H, Fang X, Li Y, Wang J, Yi B (2020). Clinical analysis of 31 cases of 2019 novel coronavirus infection in children from six provinces (autonomous region) of northern China. Zhonghua Er Ke Za Zhi.

[136] Consiglio CR, Cotugno N, Sardh F, Pou C, Amodio D, Rodriguez L, Tan Z, Zicari S, Ruggiero A, Pascucci GR (2020). The immunology of multisystem inflammatory syndrome in children with COVID-19. Cell.

[137] Riollano-Cruz M, Akkoyun E, Briceno-Brito E, Kowalsky S, Reed J, Posada R, Sordillo EM, Tosi M, Trachtman R, Paniz-Mondolfi A (2021). Multisystem inflammatory syndrome in children related to COVID-19: A New York City experience. J Med Virol.

[138] Wang M, Cao R, Zhang L, Yang X, Liu J, Xu M, Shi Z, Hu Z, Zhong W, Xiao G (2020). Remdesivir and chloroquine effectively inhibit the recently emerged novel coronavirus (2019-nCoV) in vitro. Cell Res.

[139] Wang X, Guo X, Xin Q, Pan Y, Li J, Chu Y, Feng Y, Wang Q. Neutralizing antibodies responses to SARS-CoV-2 in COVID-19 inpatients and convalescent patients. medRxiv 2020:2020.2004.2015.20065623.

[140] Duan K, Liu B, Li C, Zhang H, Yu T, Qu J, Zhou M, Chen L, Meng S, Hu Y (2020). Effectiveness of convalescent plasma therapy in severe COVID-19 patients. Proc Natl Acad Sci USA.

[141] Kolilekas L, Loverdos K, Giannakaki S, Vlassi L, Levounets A, Zervas E, Gaga M (2020). Can steroids reverse the severe COVID-19 induced "cytokine storm"?. J Med Virol.

[142] So C, Ro S, Murakami M, Imai R, Jinta T (2020). High-dose, short-term corticosteroids for ARDS caused by COVID-19: a case series. Respirol Case Rep.

[143] Yuan M, Xu X, Xia D, Tao Z, Yin W, Tan W, Hu Y, Song C (2020). Effects of corticosteroid treatment for non-severe COVID-19 pneumonia: a propensity score-based analysis. Shock (Augusta, Ga).

[144] Yang Z, Liu J, Zhou Y, Zhao X, Zhao Q, Liu J (2020). The effect of corticosteroid treatment on patients with coronavirus infection: a systematic review and meta-analysis. J Infect.

[145] Wilkinson E (2020). RECOVERY trial: the UK covid-19 study resetting expectations for clinical trials. BMJ.

